# Resolution of Lithium Deposition versus Intercalation of Graphite Anodes in Lithium Ion Batteries: An In Situ Electron Paramagnetic Resonance Study

**DOI:** 10.1002/anie.202106178

**Published:** 2021-08-13

**Authors:** Bin Wang, Lewis W. Le Fevre, Adam Brookfield, Eric J. L. McInnes, Robert A. W. Dryfe

**Affiliations:** ^1^ Department of Chemistry University of Manchester Oxford Road Manchester M13 9PL UK; ^2^ The Faraday Institution Harwell Science and Innovation Campus Quad One Didcot OX11 0RA UK; ^3^ Department of Electrical and Electronic Engineering Oxford Road Manchester M13 9PL UK; ^4^ National Graphene Institute The University of Manchester Oxford Road Manchester M13 9PL UK; ^5^ Photon Science Institute University of Manchester Oxford Road Manchester M13 9PL UK; ^6^ Henry Royce Institute for Advanced Materials University of Manchester Oxford Road Manchester M13 9PL UK

**Keywords:** electrolyte additive, graphite anodes, Li deposition, Li ion batteries

## Abstract

In situ electrochemical electron paramagnetic resonance (EPR) spectroscopy is used to understand the mixed lithiation/deposition behavior on graphite anodes during the charging process. The conductivity, degree of lithiation, and the deposition process of the graphite are reflected by the EPR spectroscopic quality factor, the spin density, and the EPR spectral change, respectively. Classical over‐charging (normally associated with potentials ≤0 V vs. Li^+^/Li) are not required for Li metal deposition onto the graphite anode: Li deposition initiates at ca. +0.04 V (vs. Li^+^/Li) when the scan rate is lowered to 0.04 mV s^−1^. The inhibition of Li deposition by vinylene carbonate (VC) additive is highlighted by the EPR results during cycling, attributed to a more mechanically flexible and polymeric SEI layer with higher ionic conductivity. A safe cut‐off potential limit of +0.05 V for the anode is suggested for high rate cycling, confirmed by the EPR response over prolonged cycling.

## Graphical abstract

An in situ EPR cell with a well‐defined electrochemical response is designed to understand the lithiation/deposition behavior occurring on the graphite anode of the Li‐ion battery. Li deposition on the graphite electrode occurs at +0.04 V (vs. Li^+^/Li) at a scan rate of 0.04 mV s^−1^. The safe cut‐off potential limit for the anode during high rate cycling and inhibition of Li deposition by a vinylene carbonate additive are revealed by in situ EPR.

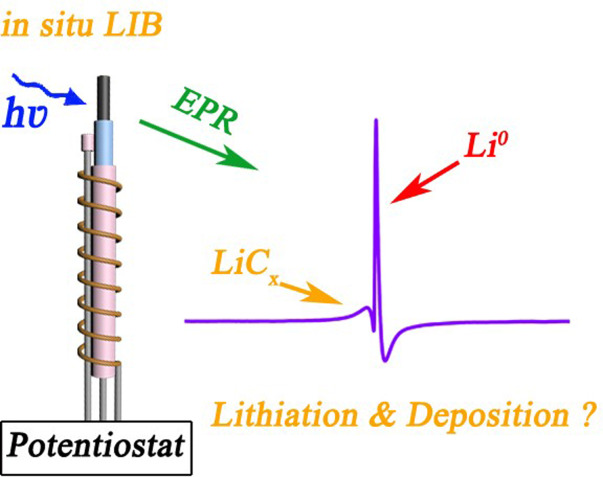

## Introduction

Li ion batteries (LIBs) play an increasingly important role in the energy system, so the development of an understanding of their degradation mechanisms is a pre‐requisite to extending battery life.^[1]^ Graphite, with a theoretical capacity of 372 mAh g^−1^, is widely used as the anode material in LIBs. Li metal deposition onto graphite can cause serious safety and degradation problems, and time‐resolved monitoring of this process is challenging.^[2]^ In common with other cell degradation processes, Li deposition can be mitigated by additives within the electrolyte, the capacity ratio between the anode and cathode, the temperature, and the charging/discharging rate.[^2a^, ^2b^, ^2c^, ^3^] Although it is normally assumed that the local potential difference at the anode/electrolyte interface has to fall below 0 V (on the Li^+^/Li scale) to overcome the overpotential associated with Li nucleation and growth, conditions most likely to be met during fast charging, recent work from Manthiram and co‐workers indicates that underpotential deposition of Li can occur during lithiation of graphite (at ca. +0.1 V vs. Li^+^/Li), triggered by dissolved Mn ions from the cathode material.^[4]^

Various non‐destructive real time techniques have been used to clarify the lithiation process and the failure mechanisms.^[5]^ In situ nuclear magnetic resonance (NMR) spectroscopy can reveal structural phase changes during lithiation via the ^7^Li frequency shift,[^5b^, ^5d^] even with early stage lithium graphite intercalation compounds (e.g. Li_1/12_C_6_).^[5b]^ Electron paramagnetic resonance (EPR) spectroscopy is a powerful alternative technique which can be harnessed to provide real time information on the chemical changes within cells, through its sensitivity to the density and environment of unpaired electronic spins. In situ EPR studies of electrochemical cells have a very long pedigree, but the technique has been surprisingly underused as a means to interrogate batteries under *operando* conditions.^[6]^ This is all the more surprising given the insights made into LIB chemistry through the application of NMR spectroscopy.[^5d^, ^5e^, ^7^] Of the notable *operando* EPR studies of LIBs to date, one report describes the Li‐rich layered oxide Li_2_Ru_0.75_Sn_0.25_O_3_ as the positive electrode, which has shown that its high capacity (>270 mAh g^−1^) is due to the reversible formation of (O_2_)^*n*−^ species.^[6c]^ However, the number of real‐time EPR studies of lithiation/de‐lithiation of carbon based anode materials is still limited.^[8]^ The first in situ EPR study of a carbon‐based material in a LIB was reported in the mid‐90’s,^[8a]^ and studies on lithiation of carbons attributed different EPR signals to “Pauli spins” (electrons at the Fermi level) and “Curie spins” (localised electrons) due to lithiation at ordered and disordered structures, respectively.[^8c^, ^9^] Wandt et al. demonstrated the power of EPR to enable time‐resolved and quantitative study of Li plating on a graphitic electrode, distinguishing between Li intercalation and deposition behaviour, in *operando* studies at −20 °C using a lithium iron phosphate counter/reference electrode.^[6i]^

In this work, a concentric geometry, three‐electrode in situ EPR cell was designed with a metallic Li cathode and graphitic anode to study (de‐)lithiation of the LIB anode at room temperature. We use this approach to assess the formation of the solid electrolyte interface (SEI) layer on the 1^st^ potential cycle, which is based on the change in microwave skin depth via its effect on the conductivity of whole cell. We further report quantitative analyses of the EPR spin density changes during multiple lithiation/de‐lithiation cycles of the graphite anode and correlate these data with electrochemical measurements on the same cell. The parallel anode processes, Li intercalation and deposition, are resolved by spectral simulation: irreversible deposition on the graphite is seen to begin at higher potentials (≥ +0.04 V vs. Li/Li^+^) even at low scan rates (0.04 mV s^−1^). The inhibition of the anode degradation by the VC electrolyte additive is confirmed by the decrease in the Li signal during long‐term cycling.

## Results and Discussion

**Figure 1:**
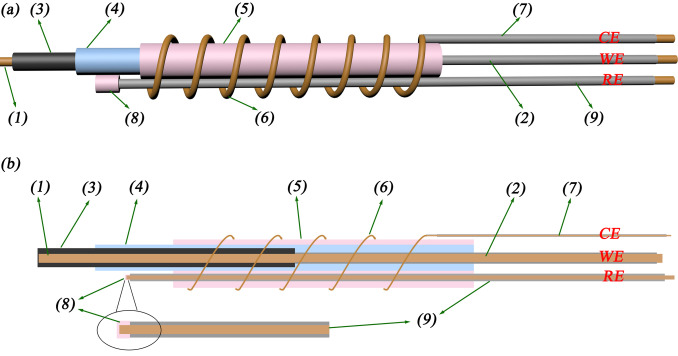
Diagram (a) and the cross‐section (b) of the three‐electrode in situ EPR cell. (1) Exposed and (2) insulated Cu wire (diameter 0.5 mm) act as the current collector for the working electrode (WE); (3) graphite anode (length 1.5 cm, mass loading ca. 0.4 mg cm^−2^, thickness 50–100 μm) coated onto the exposed part of the WE current collector (1); (4) separator (Celgard 2325, thickness 25 μm), preventing short circuit between the graphite layer and the Li; (5) Li metal layer as counter electrode (CE, length 3 cm); (6) twined, exposed Al wire (diameter 0.1 mm) and (7) insulated Al wire (diameter 0.1 mm) as the current collector for the CE; (8) Li deposited onto (9) exposed Cu wire (diameter 0.2 mm) as the reference electrode (RE); the RE is placed in the middle of the exposed part of the graphite WE.

The in situ EPR cell consisted of a three‐electrode system in a quartz tube (diameter 2 mm; Figure 1). A detailed description of the cell fabrication is given in the Experimental section (Supporting Information, Figure S1–S3).

The cell is positioned in the EPR resonator such that only the exposed graphite layer is in the sensitive part of the cavity; the Li electrode is not in the active part of the cavity (as proven by the absence of a bulk Li signal on initial set up). The concentric geometry of the in situ EPR cell gives an electrochemical performance which is close to that of the corresponding coin cell (Figure S2) without compromising on the spectroscopic sensitivity. Thus it enables study of the phase transformations of the lithiated graphite anode, and of the Li deposition process, on cycling under realistic conditions.

**Figure 2:**
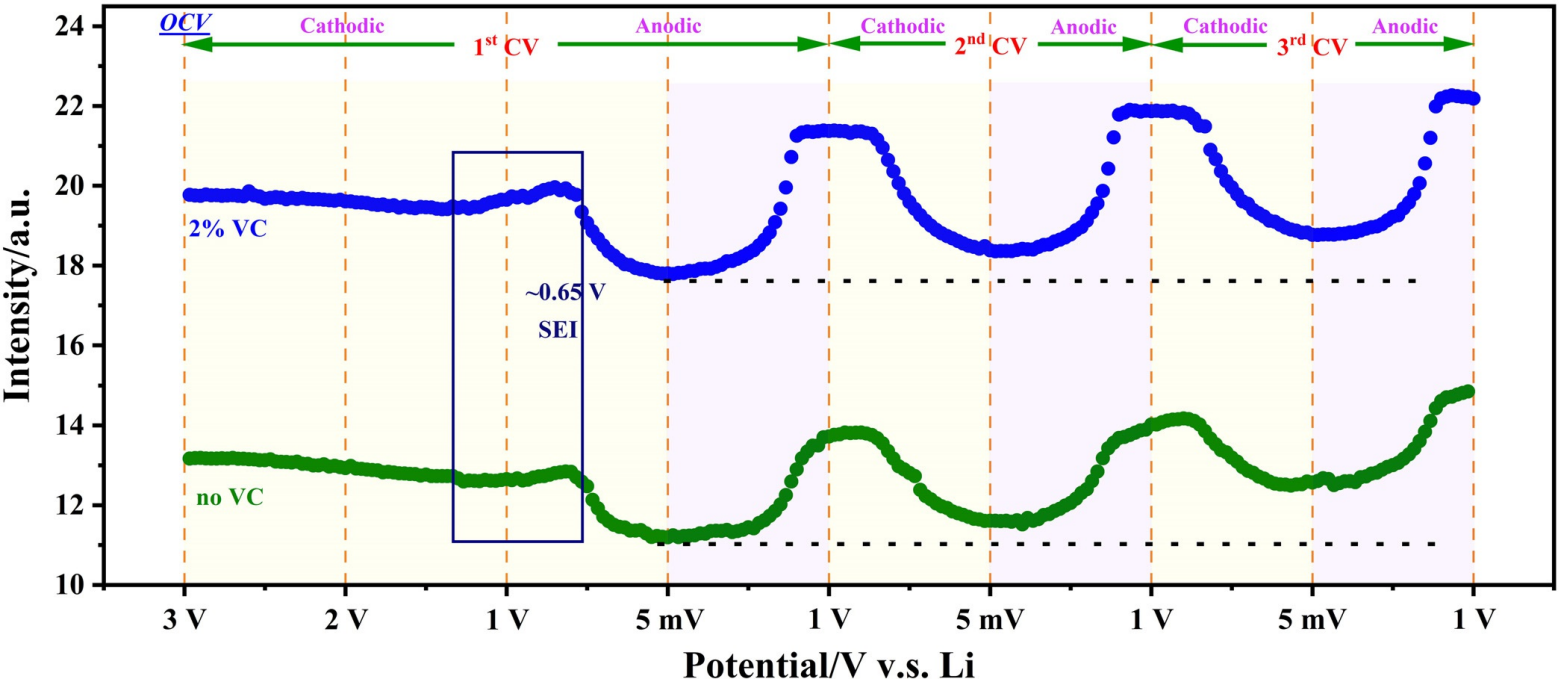
The relative intensity of the MnO standard, during the lithiation/de‐lithiation of graphite anode, with and without VC additive.

The EPR spectroscopy of conducting materials is sensitive to microwave skin depth effects,^[10]^ this has been observed in Li_*x*_C_6_ compounds as a function of lithiation.[^6i^, ^11^] The enhancement in conductivity on lithiation decreases the EPR resonator quality (or *Q*) factor and hence the EPR signal intensity. To monitor and account for this we have followed the approach outlined by Wandt et al.,^[6i]^ through use of a MnO reference sample external to the cell (detailed description in Experimental section 1.3 in SI; Figure S4a). The MnO signal intensity as a function of the applied potential (Figure S4b) reflects the changes in the resonator *Q*,^[6i]^ decreasing on sweeping to lower WE potentials, correlating with the increased lithiation and hence conductivity of Li_*x*_C_6_ (Figure 2). Hence, the maxima and minima in the MnO signal intensity (Figure 2) correlate with the discharged and fully charged states, respectively, of the graphitic electrode. We have followed this process over multiple potential cycles (Figure 2). On the first cycle, decreasing from a high starting potential, the MnO signal intensity increased slightly at around 1.3 V, and began to decrease again at around 0.65 V. Since the pristine material starts from a discharged state (also shown by the graphite EPR signal; see below), this shallow maximum in the MnO signal during the 1^st^ charging process cannot be due to de‐lithiation of the graphite. Hence, it seems likely that it can be attributed to the solid electrolyte interface (SEI) formation, which has poor electronic conductivity.^[12]^ It is also useful to exploit the evolution of *Q* to reveal the improved reversibility of the cell due to the VC additive. The SEI interface takes more time to form without the VC, which is deduced from the slower rate of change in the background response.

**Figure 3:**
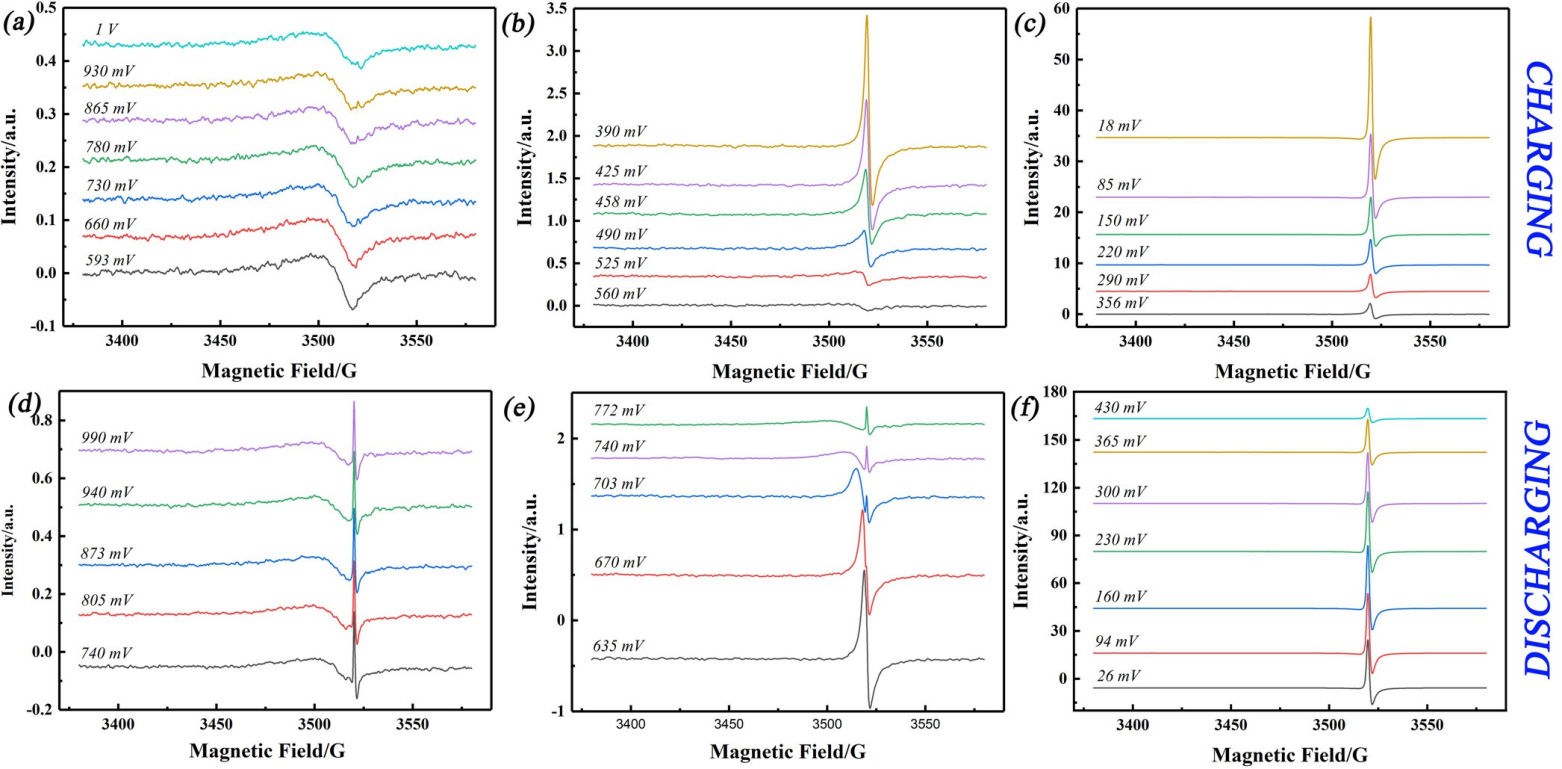
EPR signal of graphite anode at various selected potentials during charging (a)–(c) and discharging (d)–(f), during the 1st CV from +1.0 V to 0.005 V, in LP57 with 2 % VC additive.

**Figure 4:**
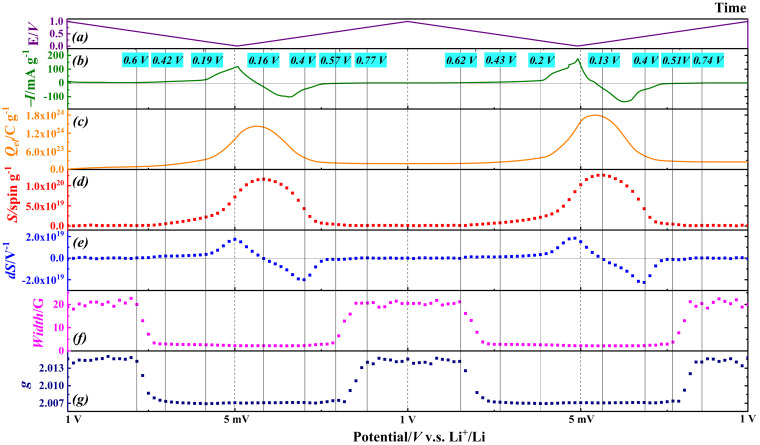
In situ EPR characterization of graphite anode in LP57 with 2 % VC. a) Potential waveform applied (*E*) to the three‐electrode cell over time. b) Current (*I*) per gram of electrode material; c) charge passed per gram (*Q*_el_) calculated from the electrochemical measurement; d) spin density per gram (*S*) calculated from the in situ EPR results; e) the 1st derivative of the spin density with respect to potential (d*S*/d*V*); f) the peak to peak linewidth and g) the *g* value of the Li_*x*_C_6_ during the first two cycles.

The EPR spectrum of the pristine graphite gives a weak signal with a Dysonian lineshape centred at *g*=2.015, indicative of the existence of mobile electrons from intrinsic defects in the graphite (peak‐to‐peak linewidth ca. 20 G; Figure 3 a).^[13]^ The spin density of the pristine graphite was calculated to be 3.79×10^17^ spins g^−1^ (see Experimental section 1.4, SI). EPR spectra were recorded every ca. 6 mins while sweeping the applied potential at 0.1 mV s^−1^ starting from 1 V to 5 mV (vs. Li/Li^+^) in LP57 (1 M LiPF_6_ in 3: 7 ethylene carbonate/ethyl methyl carbonate (EC/EMC)) with 2 % VC additive. This spectrum changes little between OCV and +0.6 V on decreasing the potential in the initial cycle (Figure 3 a). Specifically, there are no noticeable changes in the EPR spectrum as we sweep through ca. 1 V, that is, where the first peak is seen in the external MnO standard signal that monitors the resonator Q‐factor. Hence, the above deduction that the change in the MnO signal must be associated with the formation of the SEI at the graphite surface. However, we do not observe any free radical intermediates which are thought to be generated by single electron reduction of EC or the VC additive:^[14]^ these are presumably very short lived and may be detectable by spin trapping methods. Significant changes in the EPR signal are observed at potentials below ca. +0.6 V on the first sweep. Between +0.55 V and +0.42 V, a signal grows rapidly in intensity with decreasing potential, accompanied by a decrease in *g*‐value to 2.006 with a decrease in linewidth to 3 G (Figures 3 b, 4 e,f). On further decrease of the potential from +0.4 V to +0.005 V, the linewidth (2.5 G) and *g*‐value (2.006) stabilized, while the signal intensity increased rapidly (Figure 3 c). The signal could be fitted with a single Dysonian function (Figure S5 in SI),[^6i^, ^8a^] and its behavior is consistent with the formation of Li_x_C_6_ phases on charging of the cell by decreasing the potential. These changes in EPR signal were found to be reversible on increasing the potential from +0.005 to +1 V (Figure 3 d–f), as the cell is discharged. On loss of the Li_*x*_C_6_ signal at higher potentials on the first cycle, a new, narrow and very weak signal is apparent (Figure 3 d). This is attributed to the formation of Li metal and will be discussed below.[^6i^, ^15^]

Figure 4 summarizes the in situ EPR characterization of the graphite anode with 2 % VC additive in LP57, including the spin density (*S*; after calibration accounting for the changing resonator *Q* factor), the first derivative of *S* with respect to potential (d*S*/d*V*), the linewidth and the *g*‐value, charge (*Q_el_*) calculated from integration of the current recorded in the electrochemical experiment. We have correlated the EPR signal intensity (which is a measure of spin density; Figure 4 d) with the total charge passed as measured by electrochemical measurements (Figure 4 c) on the same cell. The shapes of the curves (spin density vs. charge), or their first derivatives (comparison with current data) track remarkably closely. As expected, the absolute EPR spin density is smaller than the charge injected, because EPR can only detect unpaired spin density which, in these conducting materials, are due to electrons at the Fermi level. ^[8c, 9]^ EPR is a quantitative technique for spin density, here reflecting the dynamic lithiation/de‐lithiation processes. The Li content in Li_*x*_C_6_ can be calculated from the charge number (Figure S2d): the data for the second cycle are more reliable because the first cycle involves irreversible SEI formation. The “gas type” stages (≥LiC_72_), formed at potentials higher than +0.42 V, are less readily detected by other techniques such as NMR, XRD, Raman spectroscopies due to their lower sensitivities.[^5b^, ^5c^, ^16^] In contrast, this is the regime in which we observe rapid changes in *g* and linewidth by EPR (Figures 3 and 4). Based on the charge passed, over the potential range 1.0 V to 0.42 V, we observe changes in the EPR response corresponding to an average formulation of LiC_102_ (based on the 2^nd^ CV, Figure S2d). This highlights one advantage of EPR: it is very sensitive to unpaired electrons and is blind to diamagnetic materials which will mask the spectral response in other techniques. These early stages (above +0.4 V) are thought to relate to the shallow insertion of lithium into the graphite edge. The EPR spin density increases slowly, with a constant linewidth and *g*‐value, until +0.2 V which corresponds to LiC_32_ based on the charge trace (Figure 4 b), corresponding to the formation of the stage 4 (LiC_36_).^[16]^ The spin density (Figure 4 d) increased more rapidly from +200 mV to +5 mV (corresponding to LiC_6.9_), reflecting the phase transformation of dilute stage 4 to dense stage 2/1.^[5b]^ Although we can correlate the changes in the EPR spectra with different lithiation stages, we do not observe these as discrete regions: this may be due to the limited data points over the relatively narrow potential range (200 mV to 5 mV). or possibly to the co‐existence of distinct phases as the Li^+^ penetrates further into the electrode.^[5f]^ The de‐lithiation process became faster when the potential increased from 5 mV to 0.4 V, and the complete de‐lithiation occurred at around 0.8 V as the linewidth and the g value returned back to the state of the pristine graphite. The in situ EPR data from the second potential cycle are very similar to those from the first, indicating a reversible process (Figure S6).

Results from an equivalent experiment in the absence of the VC additive in LP57 are summarized in Figure S7–9. The evolution of the signal attributed to the lithiation of the graphite anode (i.e. Li_*x*_C_6_), linewidth and *g* value, is shown in Figure S7 and S8, and was similar to the results presented above (Figure 3, 4), that is, with VC additive. By contrast, a stronger metallic Li signal was observed from the graphite anode after cycling (Figure S7 d, e), which is attributed to the irreversible formation of a much greater extent of “dead” lithium.[^2b^, ^3f^, ^6i^, ^8a^] The notable difference compared with the case of VC presence is the irreversibility of the charging process in the non‐VC case (reflected by the higher residual spin density after de‐lithiation over different cycles from Figure S9), indicating that the VC additive acts to improve the electrochemical reversibility. The EPR signal of graphite after the 1^st^ discharge process appeared significantly different (vide infra) with the strong new signal indicating the irreversible formation of degradation products generated during cycling.

As noted above, on repeated charge/discharge cycling a residual EPR signal is found in the discharged state which increases in intensity on cycling, and the lineshape simulation confirmed the existence of two different components. This signal is significantly sharper than that attributed to Li_*x*_C_6_, with a linewidth of ca. 1.25 G, and is characteristic of Li metal (Figure S10).[^6h^, ^17^] Hence, it is concluded that Li metal is deposited on the graphite at low potential even though our vertex potential is +0.005 V, as can be easily distinguished by two different EPR signals shown in Figure S11. This signal is not observable at low potentials (in the presence of VC) because the weaker Li signal is masked by the much more intense Li_x_C_6_ resonance (Figure 3). The Li metal signal has a characteristic Dysonian lineshape (Figure S12), which can be analyzed to estimate the particle size from the EPR linewidth and lineshape, specifically the A/B ratio (the peak‐to‐trough amplitude).[^6h^, ^15^, ^17^] The lineshape is due to microwave skin depth effects: particles much smaller than the skin depth give essentially isotropic signals, whereas bulk Li typically gives A/B of ca. 8 at X‐band and room temperature.[^6h^, ^6i^, ^15^, ^17^] For our cell the A/B ratio of the weak Li metal peak after de‐lithiation, in the presence of the VC additive (Figure S11a) was 1.75 after the first cycle, increasing to 2.3 after the second cycle. The increase in A/B therefore reflects the increase in size of the Li deposits for which, following Pifer's analysis,^[15]^ we estimate the mean diameters to be ca. 1.2 μm and ca. 1.6 μm after the first and second de‐lithiation cycles, respectively, assuming a skin depth of 1.1 μm at a microwave frequency of 9.8 GHz.^[17]^ Increasing the vertex potential to +25 mV led to no significant “dead” Li formation over the first three cycles, which suggests that this vertex is a safe threshold for further long cycling (Experimental section 1.5; Figure S13).

**Figure 5:**
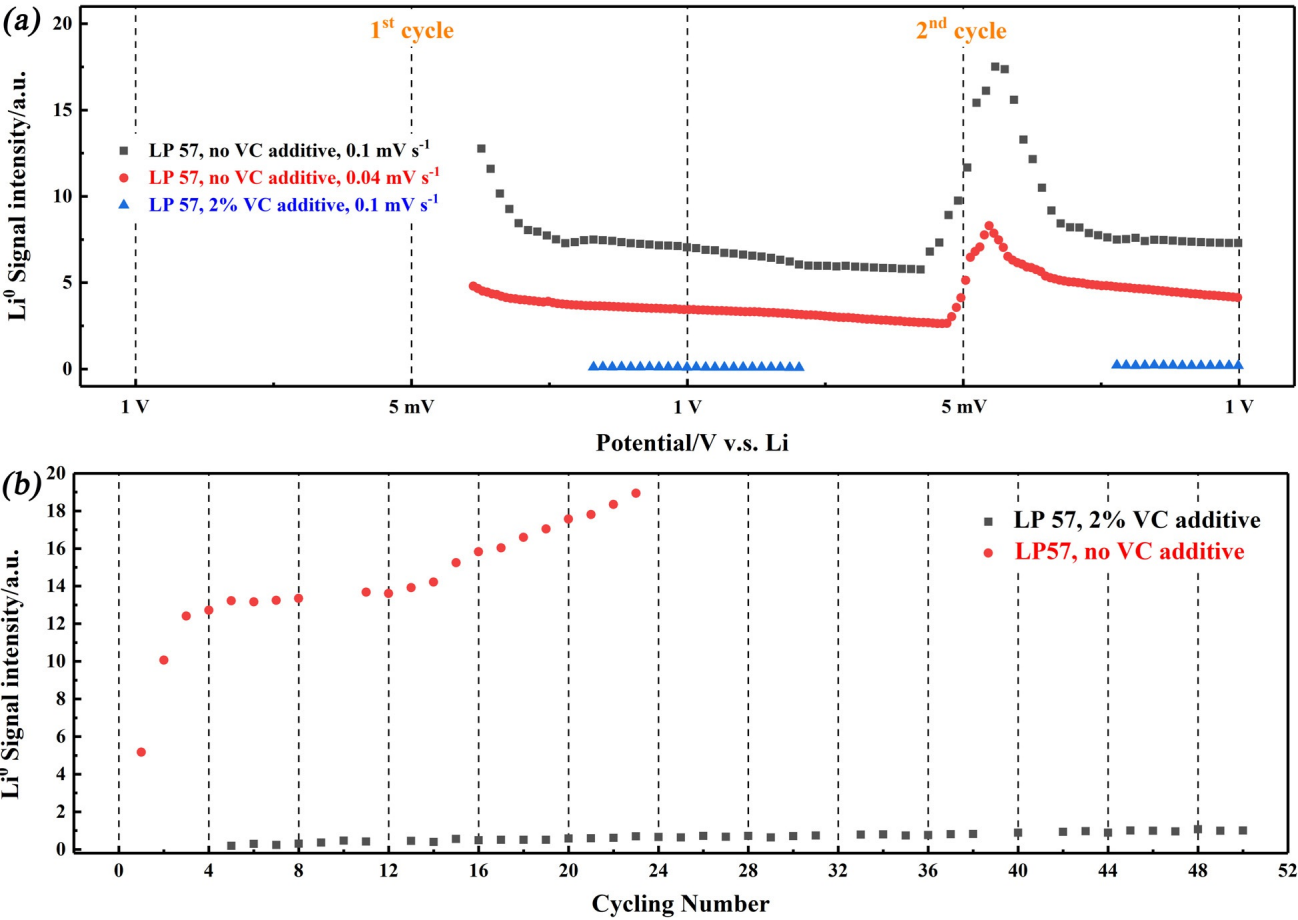
a) the EPR intensity of metallic Li^0^ deposition at graphite anode during the first two cycles with VC (blue) and without VC additive (black) at 0.1 mV s^−1^ and without VC at lower scan rate of 0.04 mV s^−1^; b) Li^0^ formation on the graphite surface during cycling from 0.05 V to 1 V at 2 mV s^−1^. The EPR signal intensity shown in (a) and (b) was normalised to the signal intensity of the pristine graphite. The missing data in (a) on the first charging cycle for all three curves, and the lower potential range for the VC result (blue curve), was due to the swamping of the weak Li^0^ signal by the Li_*x*_C_6_ at the low potential range.

In the absence of VC in the LP57, there are two notable changes, both of which indicate more rapid deposition of Li metal on the anode. First, the signal attributed to Li metal is much more intense after a given number of cycles. For example, after the first discharge sweep, the Li metal signal is an order of magnitude more intense (referenced to the intrinsic graphite defect spectrum) in the absence of VC (Figure S11b and Figure S12). Second, we find a bigger A/B≈4.5/5.5 (1^st^/2^nd^ cycle, respectively) indicating a thicker deposit, with estimated diameters of 2.3 μm and 2.5 μm. Hence, the evidence suggests the VC additive plays a significant role in suppressing the irreversible formation of Li metal deposits, which is in accord with the conclusions drawn by Dahn and co‐workers.^[3f]^ Given that the signal from the Li metal deposit is much stronger (with respect to that from Li_*x*_C_6_) in the absence of VC, it is possible to monitor it over the lower potential range (Figure 5 a, black curve). The signal cannot be detected on the initial charging sweep but (as above) it is apparent on the first discharging sweep, and it then increases in intensity after fully discharging on repeated cycling. On the first discharge, a decrease in the signal assigned to Li metal is seen, but at a slower rate of loss than the Li_x_C_6_ signal. Hence, the Li metal deposition is only partially reversible. On the second charging process, further deposition of Li metal starts when the potential is below ca. +0.1 V: the signal intensity change indicates that approximately one‐quarter of the Li deposited during the second cycle is “dead”, although this analysis does not consider the effects of changing particle size, vide infra (the corresponding EPR signal showing a response due to Li_*x*_C_6_ and Li^0^ is shown in Figure S11 b). When the scan rate is lowered to 0.04 mV s^−1^ (Figure 5 a, red curve), the deposition potential further decreases to ca. +0.05 V: for a Nernstian redox process, this potential limit would correspond to the reduction of 14 % of the lithium ions in the vicinity of the electrode. The A/B ratio of ≈2/2.9 after full de‐lithiation during the 1^st^ and 2^nd^ cycles, respectively, gives estimated particle sizes of 1.5 μm and 1.8 μm, respectively. The electrochemical stripping of deposited Li begins at a lower onset potential (e.g. <0.3 V), with a quick decrease of the Li signal. Further slow diminishing of the Li should be due to the side reaction of “dead” Li with the electrolyte. The initial increase in the Li signal during discharge may be related to the skin effect. The stripping of Li reduces the particle size, as demonstrated by a peak in the A/B value near the lower vertex potential (Figure S14). Therefore we can speculate that the EPR signal intensity continues to increase because more of the Li is becoming detectable as the particle size decreases and this initially outweighs the decrease in the total amount of Li.^[6l]^

Our results indicate that classical “over‐charging” (potentials ≤0 V vs. Li^+^/Li) is not required for Li metal deposition to occur. Heterogeneous deposition of Li, rather than homogeneous deposition on the graphite, may be more favorable.^[2c]^ The potential of the onset of Li deposition is currently debated:^[3g]^ although a value of −150 mV has been reported for deposition using a pyrolytic graphite sample,^[5g]^ a number of reports suggest deposition can occur at potentials >0 V.[^3g^, ^3h^] For example, the onset of Li deposition on a graphite anode was reported to be at +48 mV when the graphite/Li coin cell system was charged at C/100 currents at room temperature,^[18]^ From a thermodynamic perspective, this is reasonable, as the equilibrium potential, rather than the onset, of Li deposition is 0 V. From a kinetic perspective, deposition at potentials >0 V is more surprising since metal deposition normally requires an overpotential associated with the phase formation process. On the other hand, underpotential deposition of metals is also known, with recent works suggesting this phenomenon occurs in Li^+^ ion cells.^[4]^ Other factors may be at play, including the structural heterogeneity of polycrystalline graphite, which could cause local potential/current deviations. Hence it is possible that, while the net potential applied to the anode is >0 V, there is some local fluctuation in potential due to particle–particle conductivity. The properties of the SEI interface will also affect the Li deposition process: Li deposition occurs under the SEI layer and relies on the Li^+^ distribution determined by the chemical composition and the physical microstructure of the SEI layer.[^2b^, ^2c^] The VC additive helps to impart the polymeric SEI layer with increased mechanical flexibility and increased ionic conductivity.[^3f^, ^14b^, ^19^] Consequently, the current distribution on the graphite anode tends toward homogeneity and Li^+^ intercalation into graphite is more uniform. The SEI structure, which contains various inorganic Li‐based salts without the VC additive, is susceptible to breaking or cracking due to the mechanical stress induced during the lithiation/de‐lithiation process. This leads to an inhomogeneous distribution of the current density over the anode surface. Thus partial overcharging can occur, which causes subsequent Li deposition. Li deposition can occur readily as the de‐solvated ions under the SEI interface are a short distance from the graphite interface. The scan rate, 0.1 mV s^−1^, corresponds to a high C rate (3C), and is also a reason for the high potential Li deposition. Li deposition on the graphite anode is only partially reversible, that is, some “dead” lithium remained after each cycle, and there is a significant accumulation of “dead” Li after the second cycle for the cell in the absence of VC. This indicates that the VC additive can suppress the formation of metallic Li^0^ on the graphite anode and thus improve the electrochemical reversibility of the system.

The prevention of Li formation by the VC additive is also reflected in the long‐time cycling at a higher scan rate of 2 mV s^−1^ from 1 V to 0.05 V, as shown in Figure 5 b. No signal due to “dead Li” is seen during the first 4 cycles with the contribution of VC additive, only a small amount of “dead” Li is generated, which increased only slightly during further cycling. In the absence of VC, a rapid increase of “dead” Li over the initial cycles was detected on the graphite anode, followed by a weak rise and approximately linear increase with further cycling. The large increase of Li at the beginning of cycling might be related to the slowly‐formed inhomogeneous SEI interface as described above, leading to rapid deposition. The subsequent linear increase after 10 cycles is possibly due to the poor mechanical strength of the SEI without VC additive, and the associated stress during lithiation/de‐lithiation, which is likely to crack the SEI layer.

## Conclusion

In situ EPR spectroscopy has been used to understand the electrochemical behavior, including lithiation and the Li deposition, of the graphite anode upon voltammetric cycling in a bespoke three‐electrode EPR cell operated at room temperature. The EPR resonator quality factor, monitored by an external MnO standard, reflects the conductivity of the lithiated graphite and suggests that the SEI layer formation started at around 1.3 V. The EPR spectra with a narrowing process at higher potential (≥0.42 V) is correlated to the (“gas type”) stages, which are not readily detected by other techniques (e.g. NMR is typically limited to mM concentrations of spins, while μM concentrations are detectable by EPR^[20]^). The lithiated graphite (Li_*x*_C_6_, *x*<6/100) shows a single Dysonian lineshape with a constant linewidth (2.5 G) and *g* value (2.006). The spin density calculated by the EPR spectra synchronize with the evolution of the charge during cycling, and the first derivative of the spin density summarizes the phase transformation of lithiation process. The Dysonian lineshape simulation helps to separate the contribution from the Li_*x*_C_6_ and the Li deposition, assisted by the much smaller linewidth of the latter, being around 1.2 G. Further analysis reveals that the onset of Li deposition of the in situ cell occurs at higher potentials: for example, ca. +0.1 V for a scan rate of 0.1 mV s^−1^ and ca. +0.05 V for 0.04 mV s^−1^, which is mainly due to the inhomogeneous SEI layer formation. The VC additive has a significant inhibitory effect on the Li deposition during prolonged cycling, which is attributed to increased mechanical flexibility of the polymeric SEI layer compared to that formed under non‐VC conditions.

## Conflict of interest

The authors declare no conflict of interest.

## Supporting information

As a service to our authors and readers, this journal provides supporting information supplied by the authors. Such materials are peer reviewed and may be re‐organized for online delivery, but are not copy‐edited or typeset. Technical support issues arising from supporting information (other than missing files) should be addressed to the authors.

Supporting Information
