# Supplementary material for: Resolution of Lithium Deposition versus Intercalation of Graphite Anodes in Lithium Ion Batteries: An In Situ Electron Paramagnetic Resonance Study
Source: Angew Chem Int Ed Engl. 2021 Aug 13;60(40):21860–7. doi: 10.1002/anie.202106178 (PMC8518894; doi:10.1002/anie.202106178)
Supplement: Supplementary file 1 — Supporting Information [file ANIE-60-21860-s001.pdf]

Angewandte Chemie
Eine Zeitschrift der Gesellschaft Deutscher Chemiker

Supporting Information

Resolution of Lithium Deposition versus Intercalation of Graphite Anodes in Lithium Ion Batteries: An In Situ Electron Paramagnetic Resonance Study

Bin Wang, Lewis W. Le Fevre, Adam Brookfield, Eric J. L. McInnes, and Robert A. W. Dryfe**

anie_202106178_sm_miscellaneous_information.pdf

1. Experimental Section

1.1 Cell Design

Graphite powder (SPGPT806, Targary) was obtained from Targray Technology International Inc., details are shown in the below table:

Table S1, details of the graphite.[1]

	D10	D50	D90	Tap Density	Specific surface area	Pellet Density
	µm	µm	µm	g cm^{-3}	m^2 g	g cm^{-3}
SPGPT806	8.393	18.179	38.699	0.826	2.147	1.65-1.75

Graphite ink (Targray, mixed with 5 wt% carboxymethyl cellulose/styrene-butadiene rubber (CMC/SBR) 50/50 wt% binder in water) was dip-coated onto the exposed part of an insulated Cu wire (component (1), see Figure 1: diameter 0.5 mm, Advent Research Materials, UK) to form the working electrode (WE, component (2)). The morphology of the prepared working electrode was characterized by the scanning electron microscope (SEM, Hitachi SU 5000), as shown in Figure S1. A layer of separator (Celgard@ 2325, thickness 25 μm) was then rolled onto the graphite layer (part (4) in Figure 1) and dried overnight under vacuum at 80 °C. A layer of Li foil was rolled onto the separator to act as the counter electrode (CE, part (5) in Figure 1), leaving some of the graphite exposed, in the Ar-filled glovebox. The reference electrode (RE, parts (8), (9) in Figure 1) was prepared by electrodeposition of Li onto the exposed end of an insulated Cu wire (diameter 0.2 mm, Advent Research Materials Ltd., UK). All potentials are quoted with respect to this Li^+/Li RE. The RE and WE/CE were twined together by a thinner Al wire (partly exposed, diameter 0.1 mm, Advent Research Materials Ltd., UK) in the glovebox (part (6) in Figure 1). Cell materials, i.e. Cu wire, CMC/SBR, electrolyte solution, were shown to be EPR silent in control experiments, and only the graphite WE was inserted into resonant part of the spectrometer cavity. The electrolyte was LP57 (1 M $LiPF_6$ in 3: 7 ethylene carbonate/ ethyl methyl carbonate (EC/EMC)) with/without 2% vinylene carbonate (VC) additive. The concentric three electrode cell was sealed into a quartz tube (diameter 2 mm). All cell preparation steps were performed in the glove box, before transfer of the sealed cell to the EPR spectrometer.

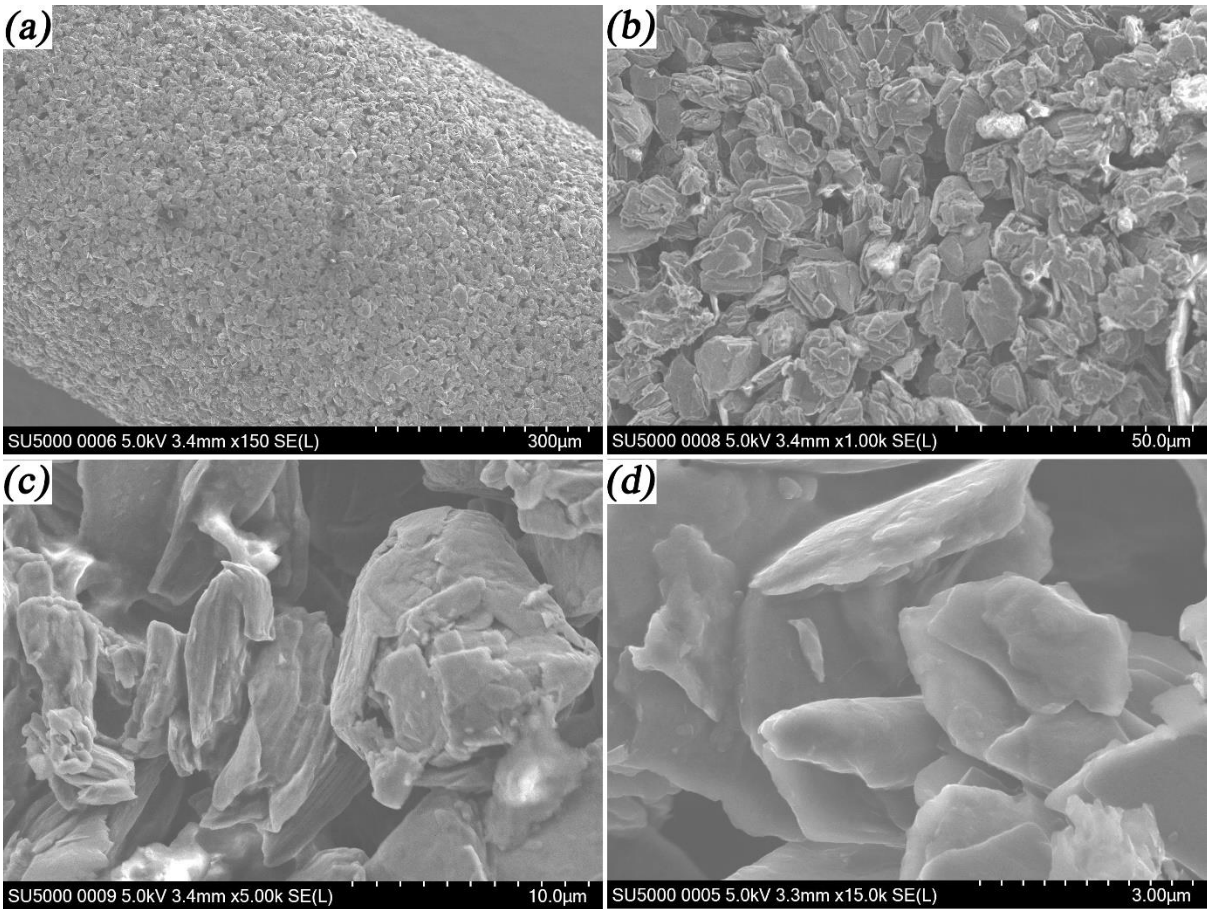

Figure S1. SEM image of the prepared working electrode, with a layer of graphite coated onto the Cu wire.

The potential distribution on the prepared cell was confirmed by the CV obtained with a standard non-aqueous redox couple, ferrocene**.** A similar cell design was used for this test, however the Li CE and RE were replaced by a Pt mesh and Al wire, respectively. The CV of the ferrocene redox couple (10 mM) is shown in Figure S2a using the graphite of the in situ EPR cell as the WE in LP57 electrolyte at a scan rate of 50 mV s^{-1}.

Figure S2b-c compare the CV of the three-electrode based coin cell (mass of the graphite anode is 2.63 mg) and the *in situ* EPR cell (the mass loading is around 1.5 mg), with a close electrochemical response. The electrochemical performance (Figure S2) was improved by minimising the WE and CE separation and the position of the RE, which was placed in the middle of the graphite roll (Figure 1). Figure S2d shows the Li content of the lithiated graphite compound as a function of cell potential. The plot was obtained by integrating the currents in the CVs as a function of the time (charge calculation) and normalizing them to Li_xC_6. The first CV gave an irreversible

capacity of around 20% during the lithiation and the de-lithiation process, which was attributed to the formation of the SEI layer. Much smaller irreversible capacity was obtained on the 2nd cycle, which was related to the continuous formation of the SEI layer during the subsequent cycles.[2, 3] The charge (Q_{el}) is obtained by integrating the currents in the CVs as a function of time.

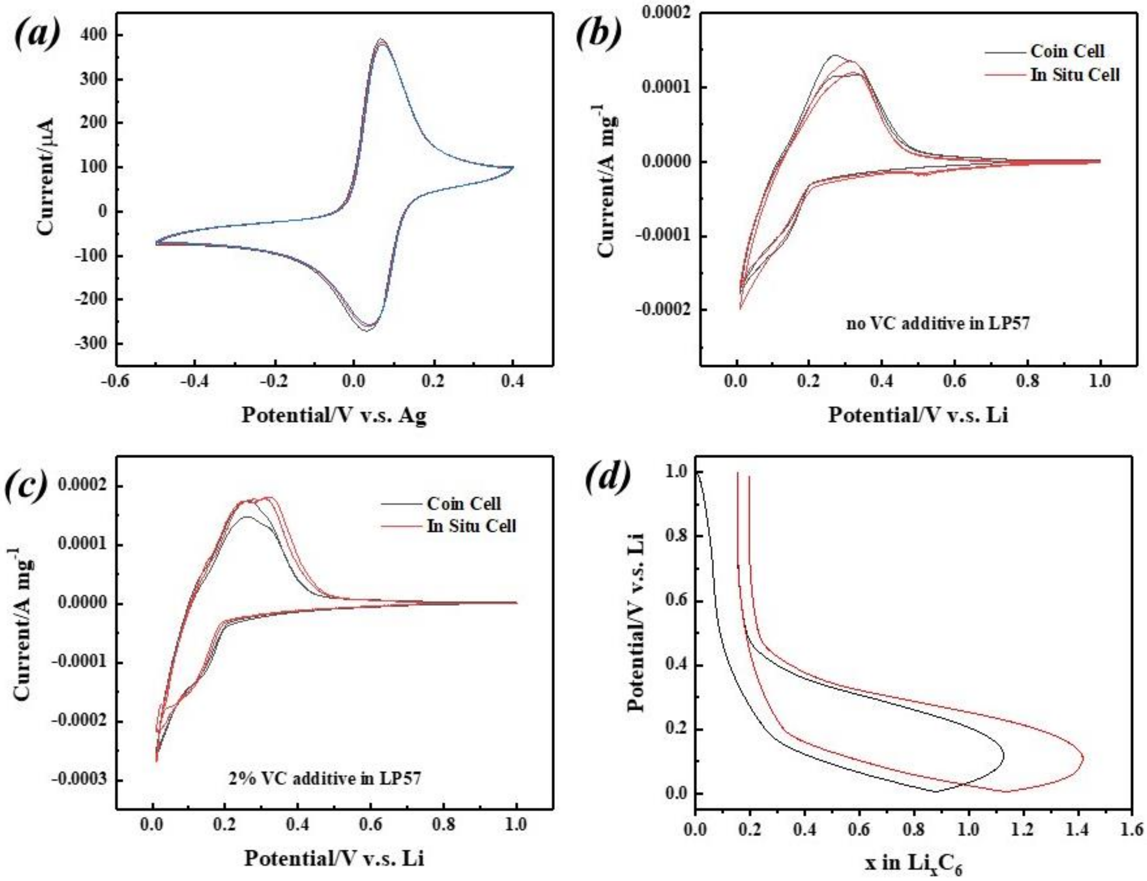

Figure S2. (a) CV for the oxidation of ferrocene in LP57 on the graphite working electrode in the in situ EPR cell at a scan rate of 50 mV s^{-1}; comparison of CV responses of the three-electrode based coin cell and the EPR cell without (b)/with (c) the VC additive; (d) The corresponding Li content, *x,* in Li_xC_6 of the in situ EPR cell in 1M LP57 with 2% VC additive (presented in Figure 4a in the main text) from 1 V to 5 mV during the 1st (black curve) and 2nd (red) curve. Mass loading is around 1-1.5 mg for the in situ cell, and 2.5 mg for the coin cell.

1.2. In situ EPR characterization

The EPR spectra were recorded at room temperature using a continuous-wave (CW) Bruker EMX Micro spectrometer. When measuring the graphite anode during lithiation process, the microwave frequency was around 9.8 GHz, the modulation amplitude was 0.5 G, the sweep width was 200 G, with a scan time of 15 s, and microwave power of 2 mW. The spectra were the average of two scans. The potential waveform was

applied with a potentiostat (EmStat^{3+} Blue, PalmSens, the Netherlands). The *in situ* EPR spectra were recorded using cyclic voltammetry at a scan rate of 0.1 mV s^{-1} from 1 V to 0.005 V. The potential change during acquisition of an individual spectrum was less than 5 mV.

The Cu wire current collector was used as the working electrode and characterized by EPR over the same potential range using the same electrolyte. The control experiment found no response from the current collector over the whole potential range.

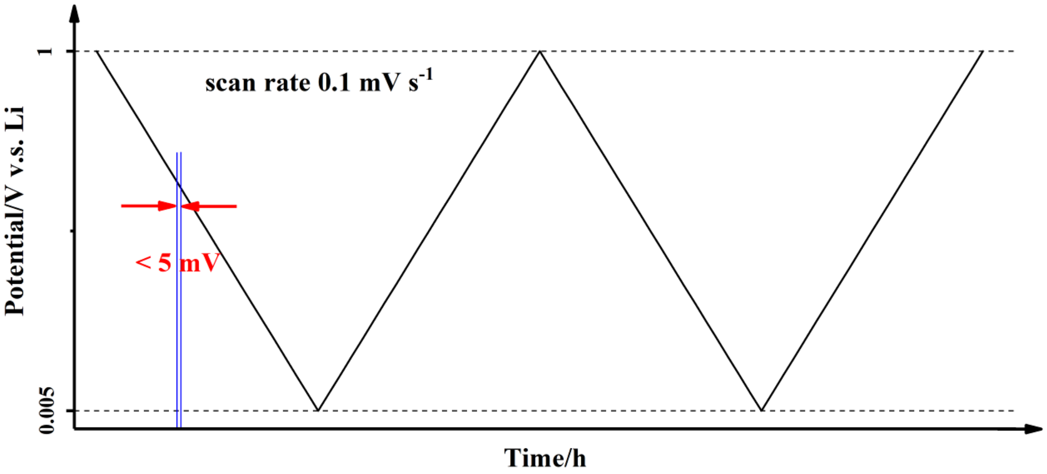

Figure S3. Scheme of the *in situ* EPR characterization under continuous CV.

1.3. Quality factor (Q) calibration

The spectroscopic quality factor (*Q*) was monitored by using an external reference material MnO outside the *in situ* EPR cell (Figure S4a).[4] For measuring the EPR signal of MnO, the microwave frequency was around 9.8 GHz, the modulation amplitude was 0.5 G, the sweep width was 1000 G, with a scan time of 15 s, and microwave power was 2 mW. The spectra were the average of two scans. The signal intensity change of MnO was related to the skin depth (δ) due to the variation in conductivity of the graphite WE on lithiation (Li_xC_6, 0≤x≤1):

$$\delta = \sqrt{1/(\sigma \pi f \mu)} \qquad \text{(Equation S1)}$$

Here, σ is the c-axis conductivity of the material, μ is the permeability and *f* is the frequency of the microwaves. Literature results suggest the conductivity (σ) of LiC_6 increases due to the lithiation process: by a factor of 10 along the a-axis (parallel to graphite sheets) and by a factor of more than 2000 along the c-axis (perpendicular to graphite sheets) compared with the pristine graphite.[4-7] As an example, the penetration conductivity of ultrathin LiC_6 graphite is reported to be 5 times higher than

$Li_{0.5}C_6$.[7] This decreased skin depth is assumed to result in a corresponding decrease in the amount of detectable graphite/MnO. The external solid state MnO powders display a nearly symmetric EPR signal at a *g* value of 2.015, with a broad linewidth of 375 G (Figure S4b).

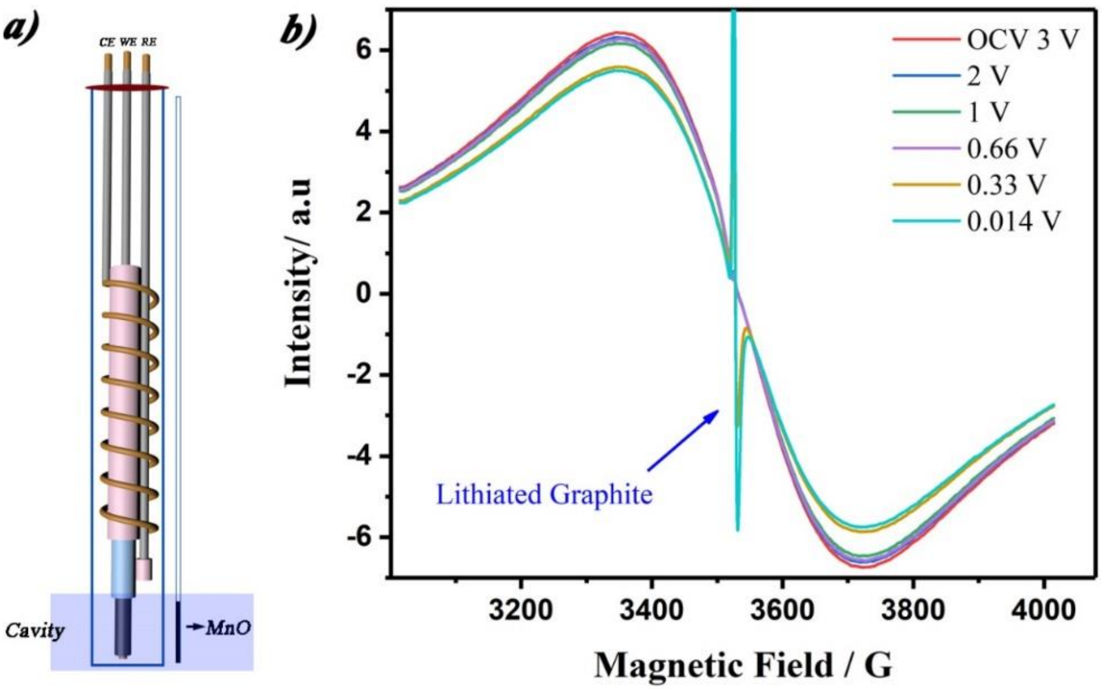

Figure S4. Scheme of the *in situ Q* factor monitoring (a) and the corresponding signal change of MnO at various potentials (b).

1.4. Spin density calculation

The spin density (*S*) of the pristine graphite was calibrated using both the standard DPPH (1,1-diphenyl-2-picrylhydrazyl)[8] and the MnO (*Q*, due to the differing conductivity of DPPH and graphite):

$$\frac{S_{Graphite}}{S_{DPPH}} = \frac{m_{DPPH}}{m_{Graphite}} \frac{DI_{Graphite}}{DI_{DPPH}} \frac{Q_{DPPH}}{Q_{Graphite}} \qquad \text{(Equation S2)}$$

Here, *m* is the mass of the sample, *DI* is the double integral of the EPR spectra, *Q* is the signal intensity of the external MnO sample. The spin density of Li_xC_6 compounds as a function of potential was calibrated by the *Q* factor.

1.5. 'inactive' Li formation during cycling

The EPR signal of 'dead' Li can only be detected at higher potentials due to the proximity of the spectroscopic *g* value of Li to that of Li_xC_6, in addition to the weak

signal intensity of the former compared to the latter at lower potentials. In order to detect the potential limit of the 'dead' Li at the graphite surface, the prepared *in situ* cell was first charged/discharged from 1 V to a successively higher vertex potential (10 mV, 20 mV, 40 mV etc.) at a scan rate of 0.1 mV s^{-1} and the EPR signal of the graphite electrode measured after several full cycles. For the *in situ* cycling experiment, the EPR signal was probed at a higher potential range (i.e. 0.9 V to 1 V) during CV (1 V to 50 mV) at the scan rate of 2 mV s^{-1}.

2. Results

2.1 Single Dysonian simulation

The single Dysonian lineshape simulation of the EPR signal of graphite at various potentials during the first charging process is shown in Figure S5. The big residual value after simulation of the plots in (a)-(c) was mainly caused by the noise, due to the weak signal of the graphite.

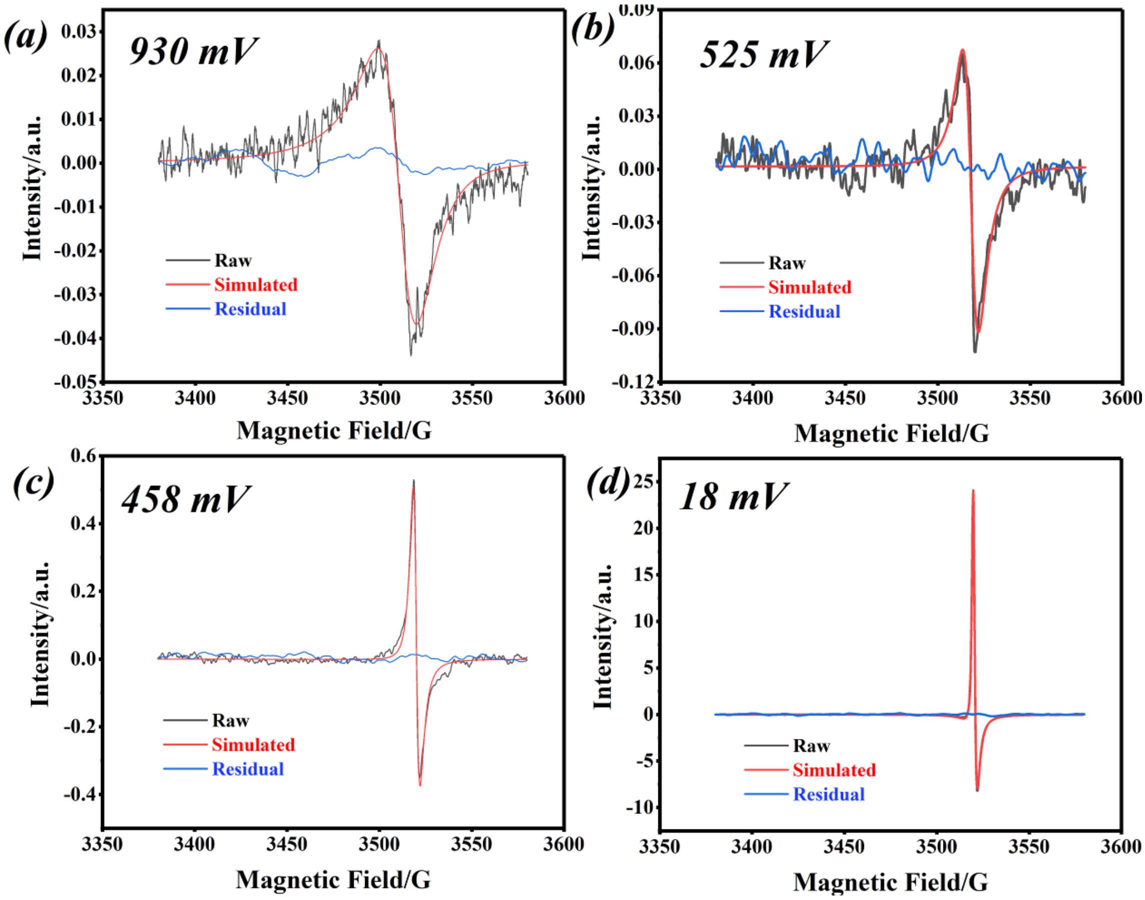

Figure S5. EPR lineshape simulation with single Dysonian function of the Li_xC_6 at different potentials during the first charging process in LP57 with 2% VC additive.

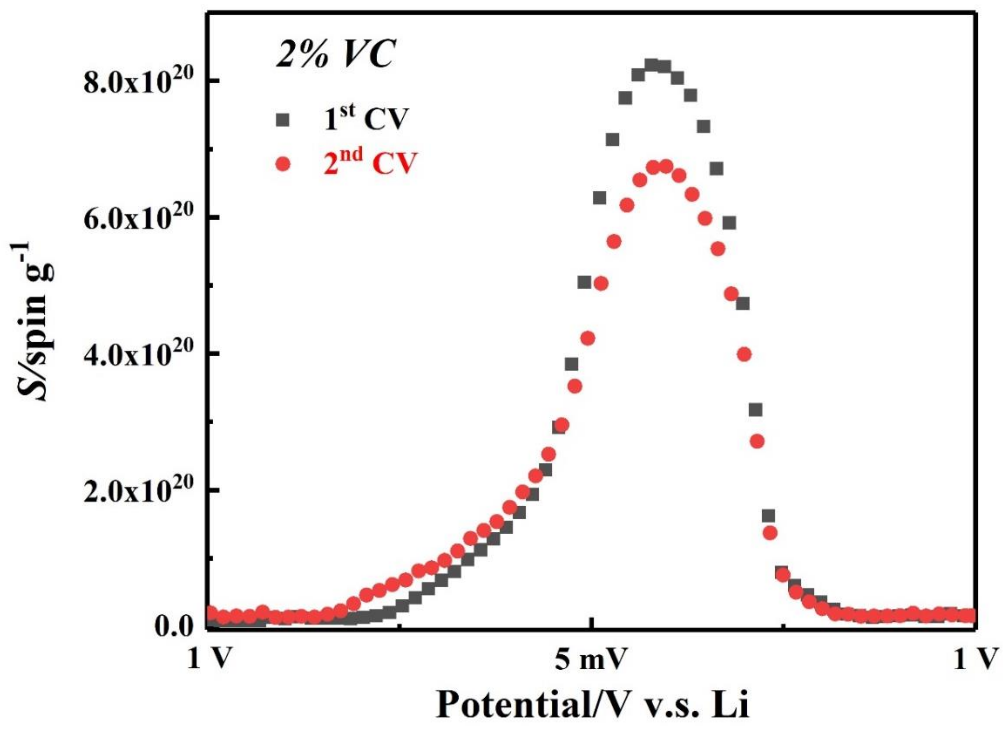

Figure S6. Spin density comparison between the 1st and the 2nd cycle of the Li_xC_6 with the VC additive present.

2.2 *In situ* EPR characterization of graphite anode in LP57.

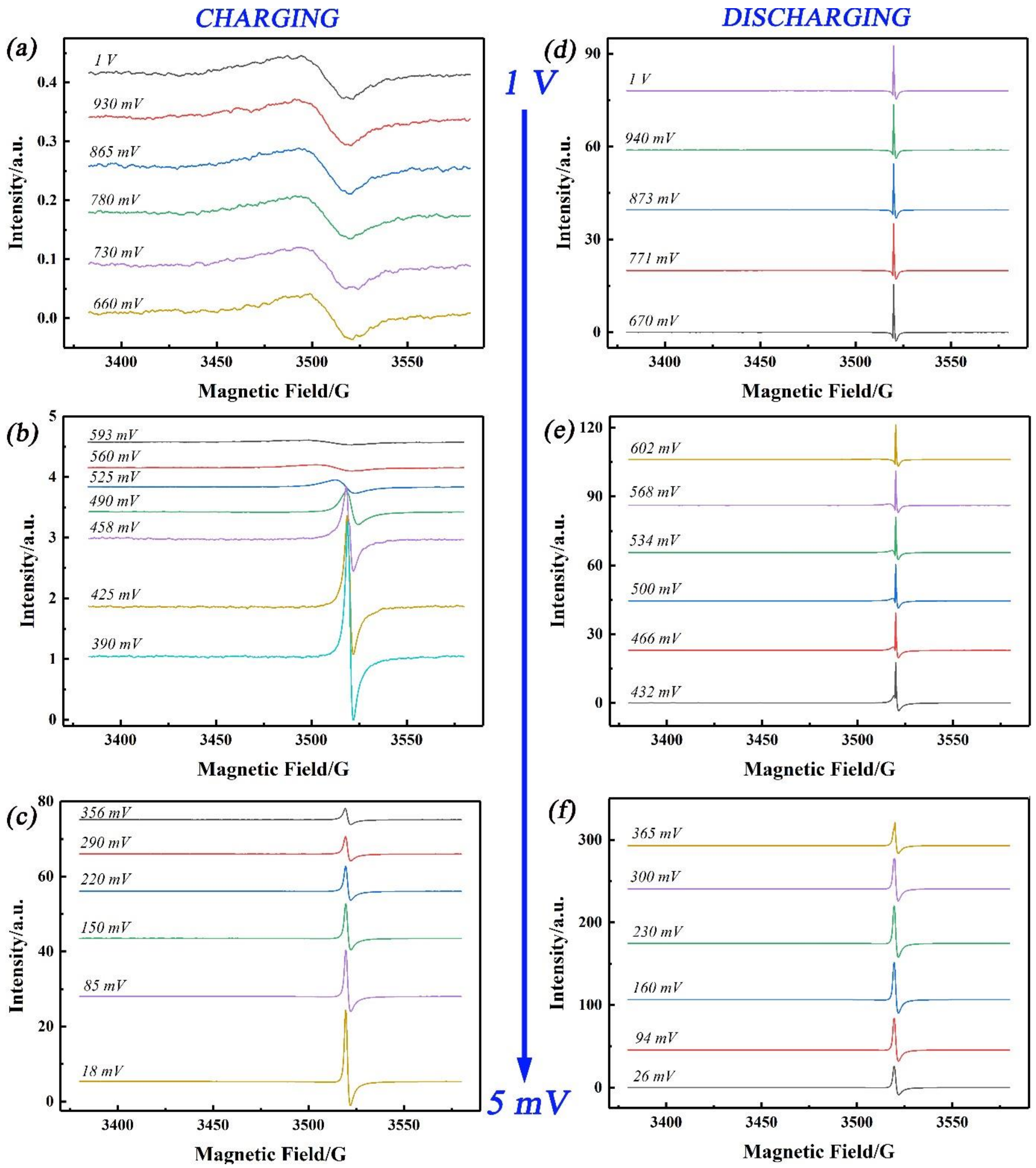

Figure S7. (a)-(c) EPR signal of graphite anode at various selected potentials during charging (a)-(c) and discharging (d)-(f), during the 1st CV from 1 V to 5 mV, in LP57 without VC additive.

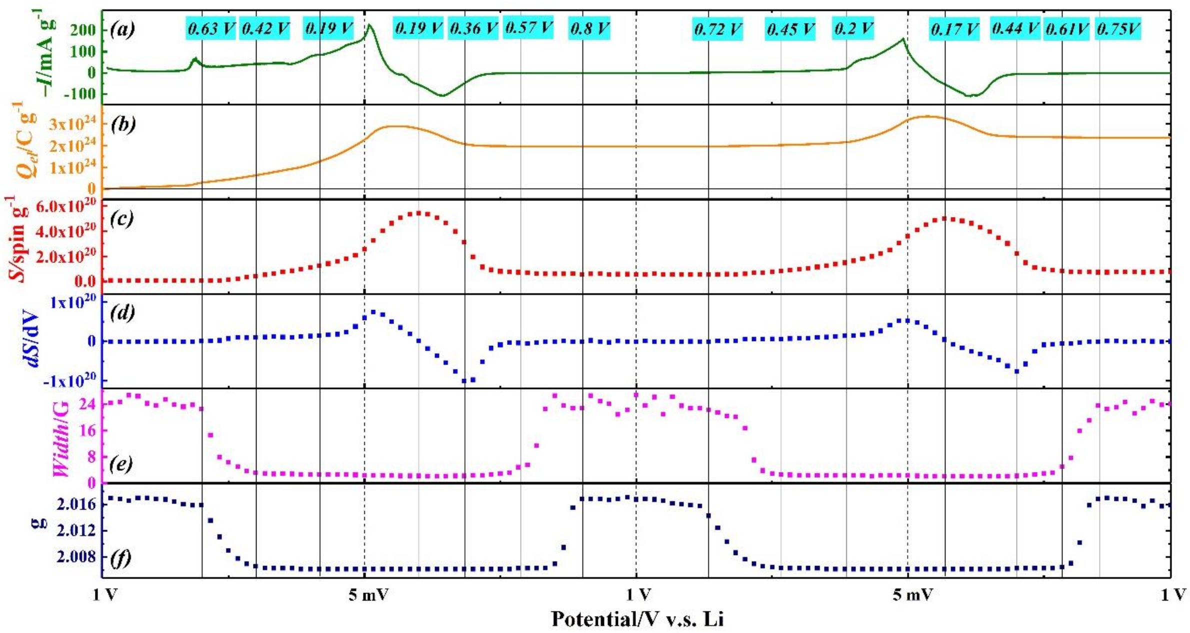

Figure S8. *In situ* EPR characterization of graphite anode in LP57 without VC additive. (a) Current per gram, (*I*); (b) charge per gram of electrode (Q_{el}) calculated from integration of the current measurement; (c) spin density per gram (*S*) calculated from the in situ EPR results; (d) the 1st derivative of the spin density with respect to potential (*dS/dV*); (e) the peak to peak linewidth and (g) the g value of the Li_xC_6 during the first two cycles.

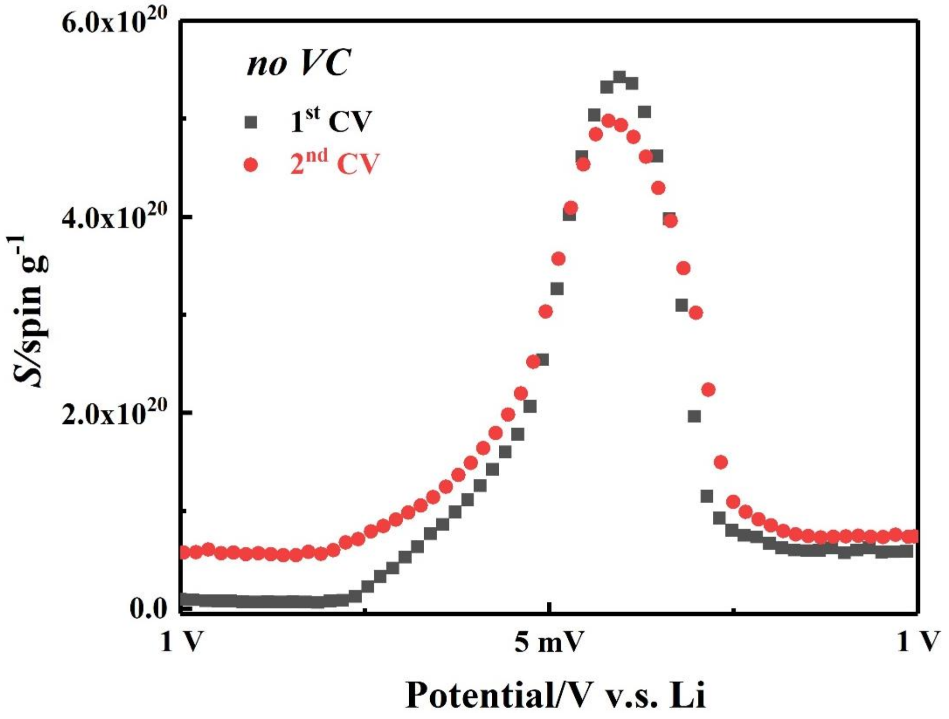

Figure S9. Spin density comparison between the 1st and the 2nd cycle of the Li_xC_6 without VC additive.

2.3 Lineshape simulation of the Li_xC_6 and the Li^0 signal

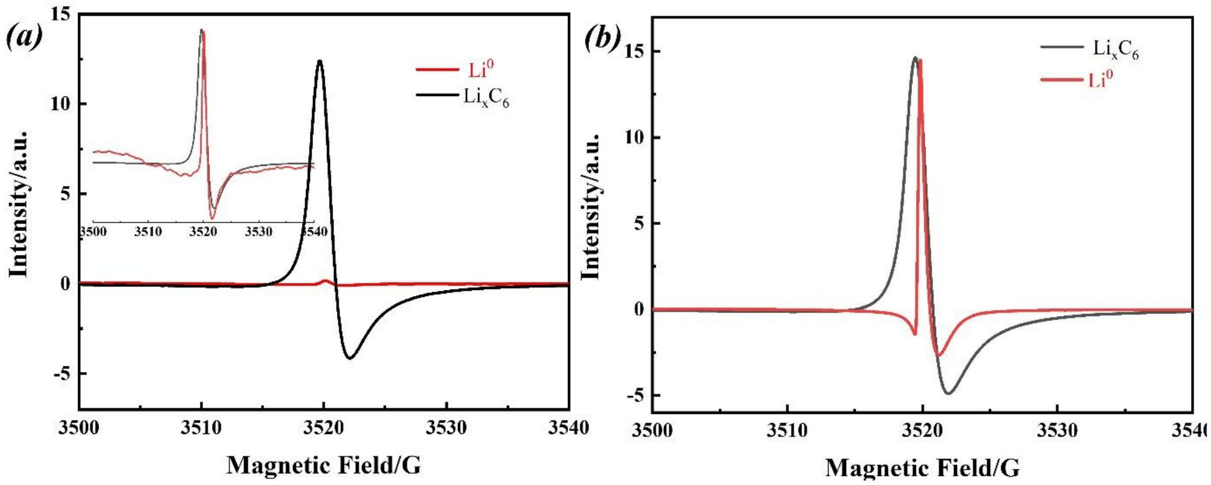

Figure S10. Comparison of the linewidth of the EPR contributions from metallic Li (black) and the Li_xC_6 (red) with the VC additive (a) and without the VC additive (b). The metallic Li signal was obtained after full de-lithiation (around 0.95 V) during the second cycle. The Li_xC_6 signal was recorded during the first charging process at a potential of 0.01 V. The signal intensity of Li_xC_6 is normalised in the inset curve in Figure a, to make the linewidth of different components comparable.

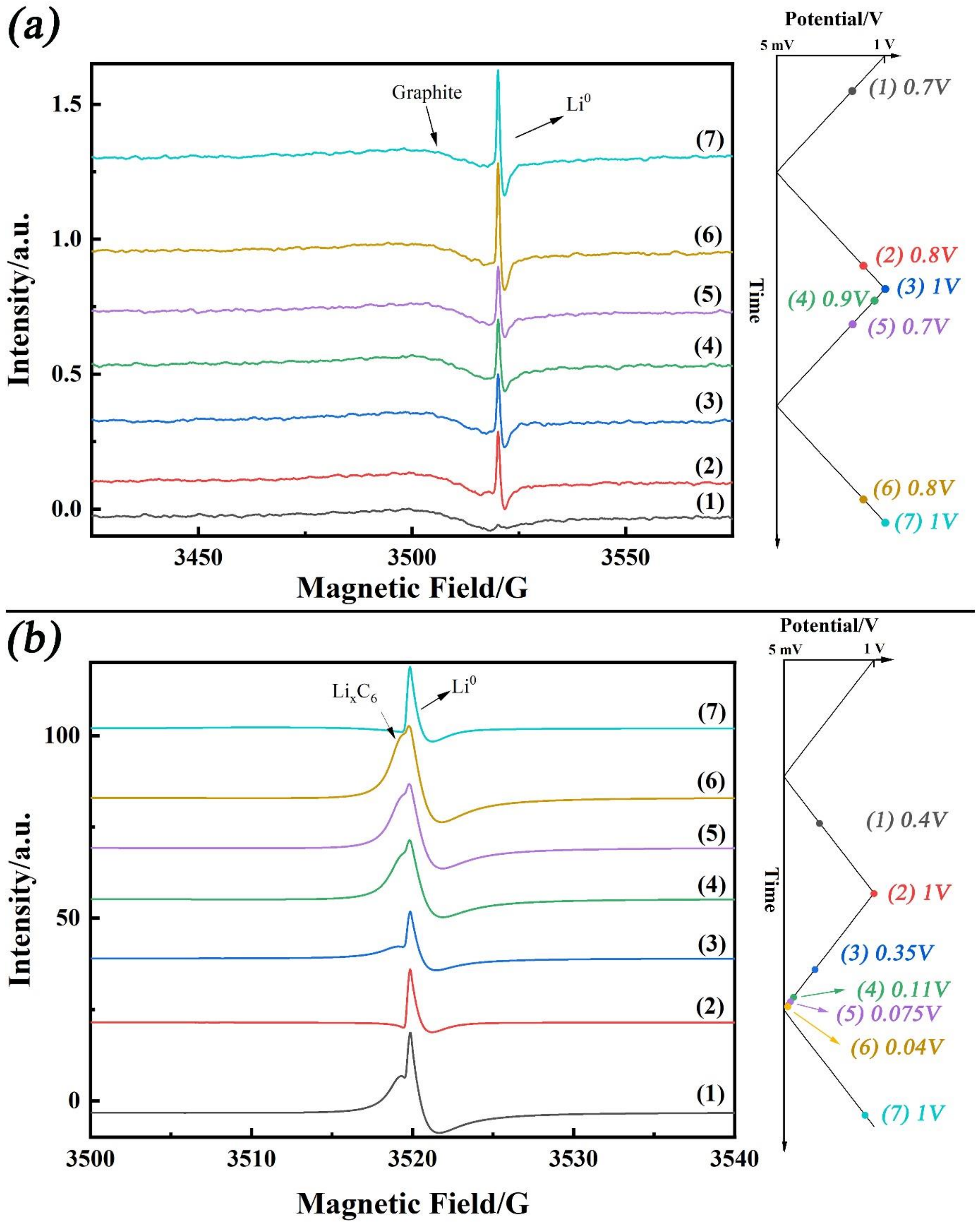

Figure S11. Selected EPR spectra of metallic Li at the graphite surface during charging with (a) VC and (b) without VC additive at the scan rate of 0.1 mV s^{-1}.

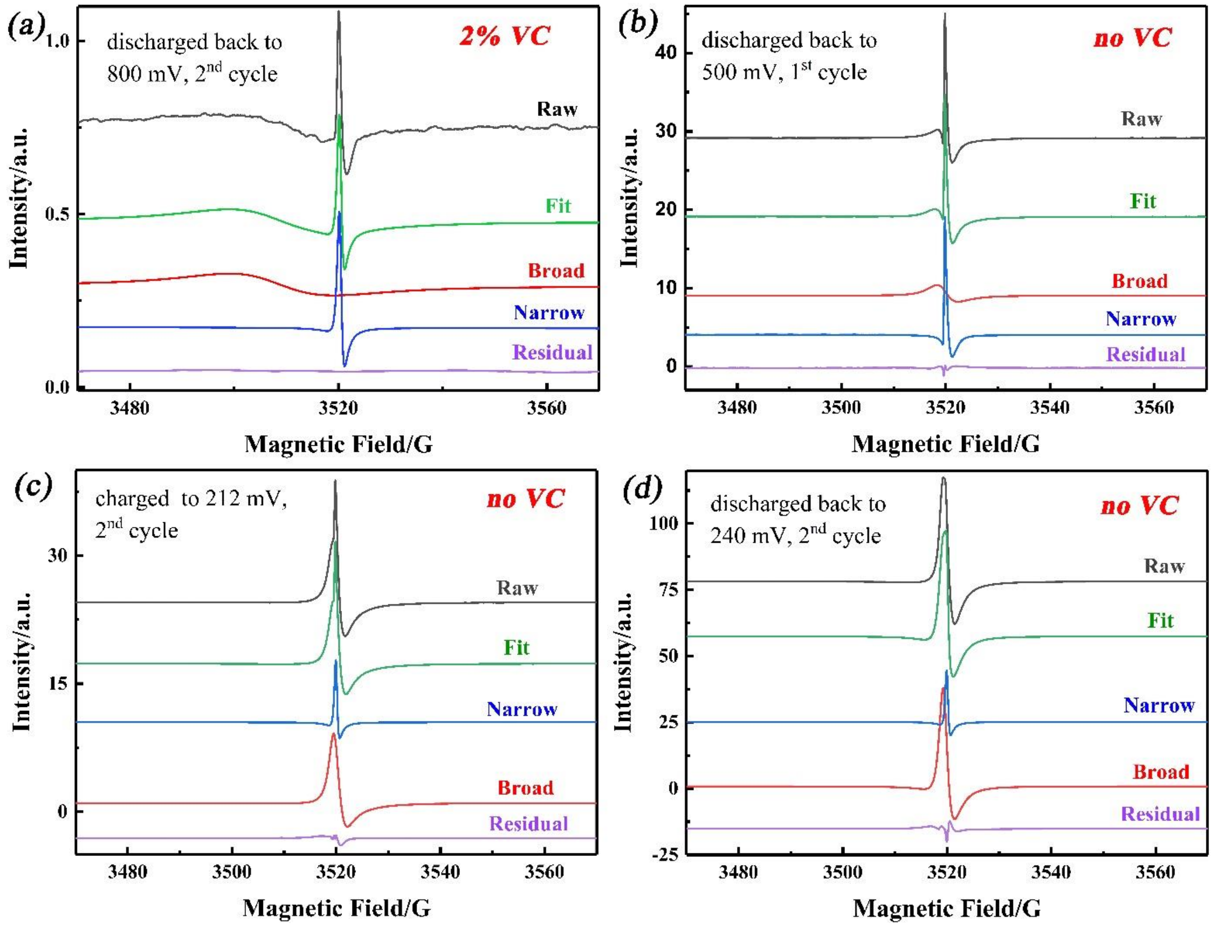

Figure S12. Examples of the two component metallic Li/Li_xC_6 simulation.

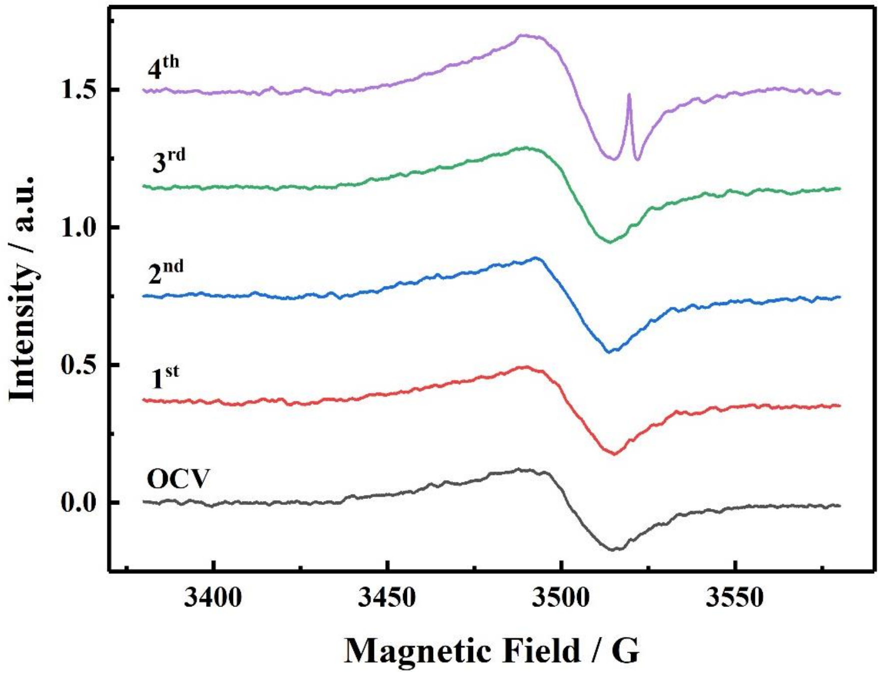

Figure S13. EPR signal of the graphite anode after full de-lithiation for several cycles at 0.1 mV s^{-1} between 1 V and + 25 mV.

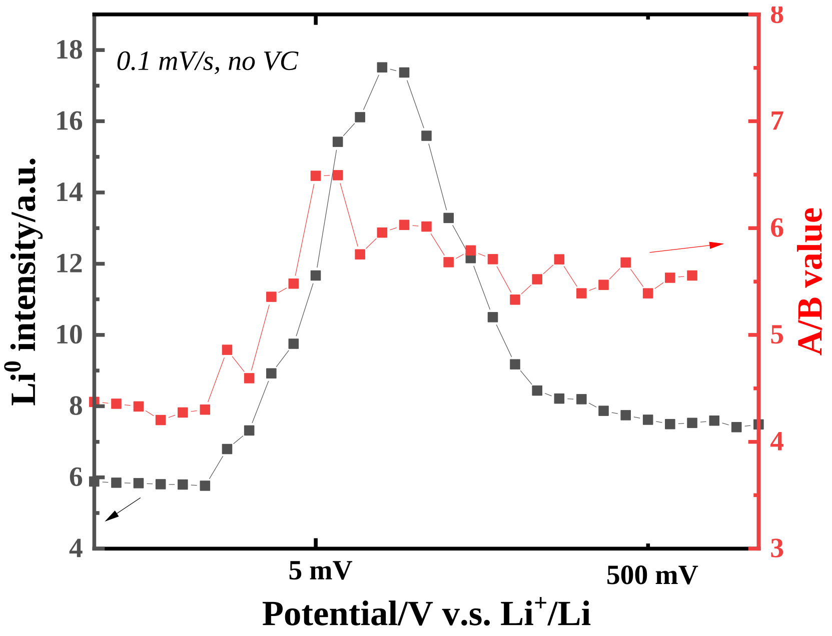

Figure S14. The A/B value change of the Li^0 signal during charging/discharging process in LP 57 electrolyte without VC additive at the scan rate of 0.1 mV s^{-1}.

The asymmetry factor (A/B value) of a Dysonian lineshape can be used to characterize the size of the Li particles.[9] The increased signal intensity of Li^0 during the initial discharge process does not necessarily correspond to an increase in the total quantity of metallic Li during stripping. A high A/B value suggests the deposited Li has a larger particle size than the skin depth. More EPR detectable skin volume may thus be exposed during the stripping of large Li particles (as the roughness of Li surface can change) when the cell is discharged. This is consistent with the change in the A/B value shown in Figure S14, in which the A/B value reaches a maximum near the lowest vertex potential. A lower A/B suggests a smaller particle size. Similar results are found in the recent paper by Dutoit *et. al*.[10]

References (Supporting Information):

1. Data from the supplier: https://www.targray.com/li-ion-battery/anode-materials/graphite
2. Smith, A. J.; Burns, J. C.; Zhao, X.; Xiong, D.; Dahn, J. R., A High Precision Coulometry Study of the SEI Growth in Li/Graphite Cells. *J. Electrochem. Soc.* **2011,** *158* (5), A447.
3. McArthur, M. A.; Trussler, S.; Dahn, J. R., In Situ Investigations of SEI Layer Growth on Electrode Materials for Lithium-Ion Batteries Using Spectroscopic Ellipsometry. *J. Electrochem. Soc.* **2012,** *159* (3), A198-A207.
4. Wandt, J.; Jakes, P.; Granwehr, J.; Eichel, R.-A.; Gasteiger, H. A., Quantitative and time-resolved detection of lithium plating on graphite anodes in lithium ion batteries. *Mater. Today* **2018,** *21* (3), 231-240.

5. Dresselhaus, M. S.; Dresselhaus, G., Intercalation compounds of graphite. *Adv. Phys.* **1981,** *30* (2), 139-326.
6. Basu, S.; Zeller, C.; Flanders, P. J.; Fuerst, C. D.; Johnson, W. D.; Fischer, J. E., Synthesis and properties of lithium-graphite intercalation compounds. *Mater. Sci. Eng.* **1979,** *38* (3), 275-283.
7. Bao, W.; Wan, J.; Han, X.; Cai, X.; Zhu, H.; Kim, D.; Ma, D.; Xu, Y.; Munday, J. N.; Drew, H. D.; Fuhrer, M. S.; Hu, L., Approaching the limits of transparency and conductivity in graphitic materials through lithium intercalation. *Nat. Commun.* **2014,** *5* (1), 4224.
8. Yordanov, N. D.; Christova, A., DPPH as a primary standard for quantitative EPR spectrometry. *Appl. Magn. Reson.* **1994,** *6* (1), 341-345.
9. J. H. Pifer, R. Magno, *Phys. Rev. B* **1971**, *3*, 663-673.
10. C.-E. Dutoit, M. Tang, D. Gourier, J.-M. Tarascon, H. Vezin, E. Salager, *Nat. Commun.* **2021**, *12*, 1410.
